# PERspectives on circadian cell biology

**DOI:** 10.1098/rstb.2023.0483

**Published:** 2025-01-23

**Authors:** Andrei Mihut, John S. O'Neill, Carrie L. Partch, Priya Crosby

**Affiliations:** ^1^ MRC Laboratory of Molecular Biology, Francis Crick Avenue, Cambridge CB2 0QH, UK; ^2^ Department of Chemistry and Biochemistry, University of California, 1156 High Street, Santa Cruz, CA 95064, USA; ^3^ Institute of Cell Biology, School of Biological Sciences, University of Edinburgh, Edinburgh EH9 3BF, UK

**Keywords:** circadian rhythm, cellular clock, post-translational modification, intrinsically disordered regions (IDRs), PERIOD/ PER, biomolecular condensation/ phase separation

## Abstract

Daily rhythms in the activities of PERIOD proteins are critical to the temporal regulation of mammalian physiology. While the molecular partners and genetic circuits that allow PERIOD to effect auto-repression and regulate transcriptional programmes are increasingly well understood, comprehension of the time-resolved mechanisms that allow PERIOD to conduct this daily dance is incomplete. Here, we consider the character and controversies of this central mammalian clock protein with a focus on its intrinsically disordered nature.

This article is part of the Theo Murphy meeting issue ‘Circadian rhythms in infection and immunity’.

## 1. Introduction: the functionality of temporal organization

Life on Earth, in all its complex diversity, must contend with significant fluctuations in light, temperature and nutrient availability resulting from the 24 hours rotation of our planet around its axis. To maximize their prospects of survival in this dynamic environment, most organisms have developed daily timing strategies. These circadian rhythms (term derived from the Latin ‘*circa*’, meaning ‘around’ and ‘*dies*’ meaning ‘day’) have evolved to provide an adaptive advantage, enabling organisms to anticipate and synchronize their biological processes with daily environmental cycles [1,2]. Critically, circadian timekeeping is endogenous and so persists even in the absence of external stimuli. Circadian rhythmicity is observed in most animals, algae and plants, as well as many fungi, suggesting it was likely present in early eukaryotes [3], and is also observed in photosynthetic and a few non-photosynthetic prokaryotes, further emphasizing its broad biological significance [4].

In humans, circadian oscillations are found throughout physiology, from brain and body temperature [5] to blood pressure and metabolism, hormone levels (e.g. melatonin, cortisol and insulin) and the sleep–wake cycle [6–8]. Circadian disruptions have significant impacts upon human health and disease; the chronic circadian dysregulation experienced by long‐term shift workers is strongly associated with a range of diseases such as type II diabetes, cardiovascular disease, neurodegenerative disorders [9] and various forms of cancer [10,11]. Thus, circadian regulation pervades healthy human physiology, whereas circadian dysregulation contributes to disease [12].

The earliest recorded description of diurnal oscillations comes from Androsthenes of Thasos in the fourth-century BCE, who noted the daily movements of tamarind tree leaves. Almost 2000 years later, the first truly circadian rhythm was documented in 1729 by French physicist Jean-Jacques d’Ortus de Mairan, who described the rhythmic opening and closing of the leaves of *Mimosa pudica*, which persisted in constant darkness (demonstrating the endogenous nature of circadian timekeeping). Since then, circadian oscillations have been discovered across the kingdoms of life [3], for instance in the pupal eclosion of *Drosophila*, and the wheel running activity of mice [13].

Several key fundamental features define a circadian oscillation [14]: it must be endogenous, self-sustaining and persist in the absence of any external cues with an approximately 24 hour period; the phase of the oscillation must be capable of being synchronized by external timing stimuli, such as light–dark, feeding and temperature cycles [15,16] and the period length of the oscillation must be temperature compensated, retaining its periodicity across a range of biologically relevant temperatures [17,18].

In mammals, lesion studies performed in the brain identified a region in the hypothalamus which was shown to be essential for generating and maintaining circadian rhythmicity in physiology and behaviour. This suprachiasmatic nucleus (SCN) represents a network of approx. 20 000 highly interconnected cells, which coordinates circadian physiology through neuronal and hormonal signals [19–21]. Despite the central role the SCN plays at a whole organism level, the fundamental basis of circadian rhythmicity is the cell, as circadian oscillations are observed in peripheral tissues and persist *ex vivo*, in isolated tissues and cells in a dish [22–24]. This cell-autonomous function is highlighted by numerous studies showcasing circadian physiology such as cellular responses to viral infections [25], cellular motility [26], electrical activity in cardiomyocytes [27,28] and phagocytic activity [29]. Even the most fundamental functions of mammalian cells show circadian regulation in culture, for example, translation rate, primary metabolic flux and even the timing of cell division [30–32].

## 2. Existing model for circadian regulation of cell function

**Figure 1.**
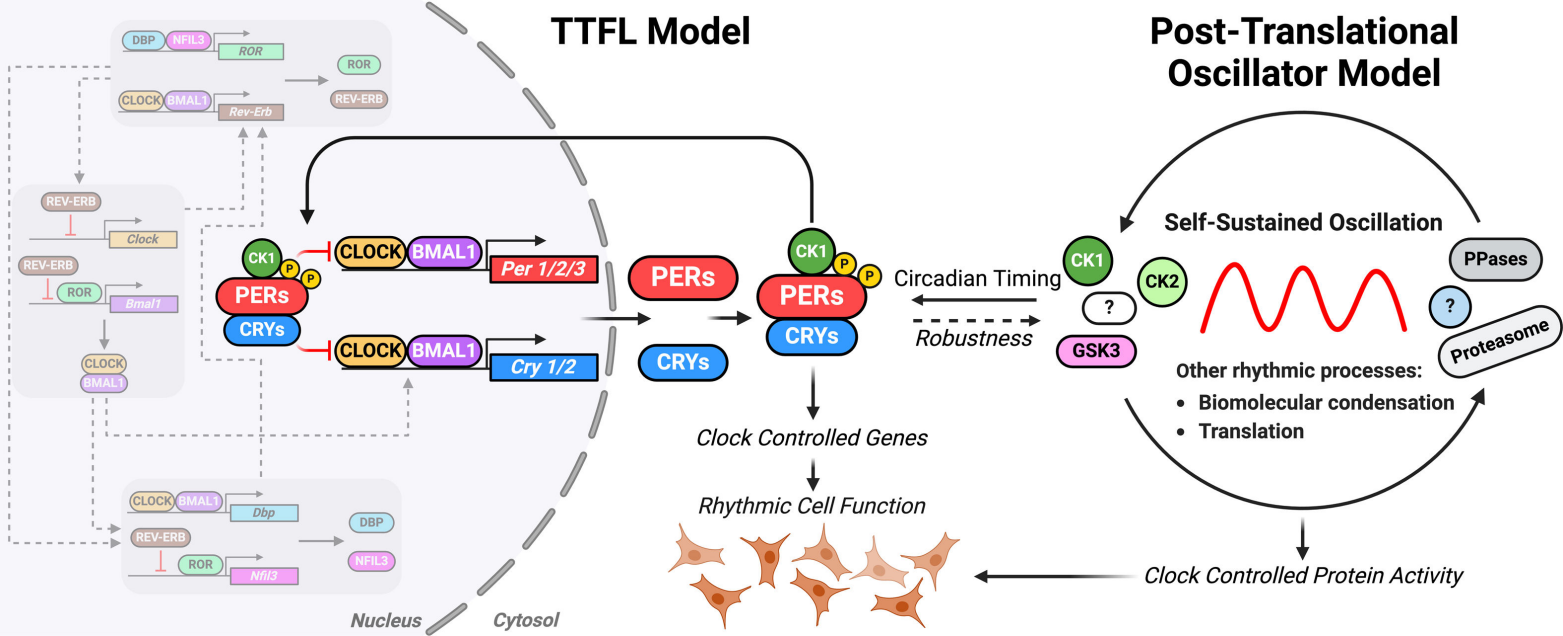
Schematic representation of models for circadian rhythm generation. The TTFL model for cellular circadian timekeeping: it intrinsically generates and sustains oscillations by itself, with a post-translational delay-timer being responsible for conferring the approx. 24 hour periodicity. It imparts rhythmic cellular functions by directly regulating the transcription of clock-controlled genes. On the other hand, the post-translational oscillator model proposes a post-translational timekeeping mechanism capable of autonomously generating and sustaining approx. 24 hour oscillations in protein activity on its own. It conveys timing information to the TTFL, which acts as a signal transducer, via post-translational modifications of TTFL components to regulate the transcription of TTFL genes and downstream clock-controlled genes. While the post-translational oscillator confers timing information to the TTFL, the latter may confer robustness in return, by further amplifying the timing information received and by regulating enzyme activity directly via interactions with TTFL proteins. Figure created with BioRender.com.

Significant work performed over the past 50 years has led to the development of a molecular model for the generation of circadian rhythms that relies on the delayed transcriptional feedback repression of ‘clock genes’ that regulate their own expression through a transcriptional-translation feedback loop (TTFL), as well as that of many downstream ‘clock-controlled genes’ that go on to regulate wide-ranging circadian physiology. Elucidation of the central TTFL circuit in *Drosophila melanogaster* provided the template for TTFLs in other metazoa and was recognized by a Nobel Prize in 2017. In the canonical mammalian TTFL (figure 1), which has been extensively reviewed previously [33], transcriptional activators Circadian Locomotor Output Cycles Kaput (CLOCK) and Brain and Muscle ARNT-Like 1 (BMAL1, also known as ARNTL1) function as a heterodimeric pioneer factor that binds to E-box elements in the promoters of numerous genes, most notably *Period* (PER, which has three paralogs: PER1, PER2 and PER3) and *Cryptochrome* (CRY, which has two paralogs: CRY1 and CRY2). The encoded PER and CRY proteins, together with casein kinase 1 (CK1), form an ‘early’ repressive complex in the cytosol that translocates into the nucleus where it suppresses the activity of CLOCK·BMAL1, thereby inhibiting their own activation. PER and CRY are subsequently degraded, but sufficient CRY1 remains to continue repressing transcriptional activation for some hours more in the ‘late’ repressive complex [34]. Only once CRY1 has also been degraded does the approx. 24 hour cycle restart.

Collectively, these genes are referred to as the ‘core clock genes’, a rather loose term which is generally used to refer to a gene whose expression is involved in the mechanism of circadian timekeeping to elicit an approx. 24 hour oscillation [35]. Further regulation is imparted through various auxiliary feedback loops, aiding in the broader regulation of rhythmic gene expression. One such example involves the nuclear receptors REV-ERBα and β, encoded by the genes Nr1d1 and Nr1d2, respectively, and retinoic acid receptor-related orphan receptors (ROR, which includes RORα, RORβ and RORγ) which are transcriptionally activated by CLOCK·BMAL1 and in turn inhibit *Bmal1* transcription to produce a *Bmal1* transcript oscillation that is anti-phasic with that of PER protein production (figure 1) [36–38]. An additional feedback loop involves the D-box binding protein (DBP), a transcriptional activator which binds to D-box promoter elements and opposes the activity of the repressor nuclear factor interlukin-3 regulated, which is itself regulated via the REV-ERB/ROR loop [33] (figure 1). Overall, while these auxiliary feedback loops contribute to the regulation of downstream clock-controlled genes, they are not essential for generating circadian rhythmicity in cells.

The mammalian TTFL circuitry is similar across Metazoa [39] and mirrored in other eukaryotic kingdoms. For instance, the fungus *Neurospora crassa* makes use of a functional analogue of PER named Frequency (FRQ), which inhibits its own transcription [40]. However, while the underlying form of circadian transcriptional regulation appears preserved across eukaryotes, the identity of the specific transcriptional effectors is not. It has therefore been suggested that these mechanisms evolved multiple times, independently, through convergent evolution, rather than from a common ancestor [41].

## 3. Limitations of the current model

The TTFL model was proposed more than 30 years ago [42]. For mammalian cells, it provides a very convenient, simple and clear explanation for both the molecular basis of circadian rhythm generation and also physiological regulation, via clock-controlled gene transcription. The evidence that circadian TTFLs occur in most mammalian cell types under physiological conditions *in vitro* and *in vivo* is overwhelming, as is the evidence that circadian physiology is profoundly disrupted when paralogous TTFL genes are deleted, e.g. Bmal1^−/−^, Cry1^−/−^/2^−/−^or Per1^−/−^/2^−/−^ [43]. However, the direct evidence that any TTFL is necessary and sufficient to generate a circadian rhythm is less clear cut. For example, based on our current understanding of the model, rhythms should be abolished by constitutive expression of *Per* or *Cry*, but are not [44–46]. This, taken together with several other observations discussed in detail below, suggests that there may now be sufficient accumulated evidence to warrant a revision of the precise molecular timekeeping mechanism and how this achieves daily organization of physiology.

A major limitation of the TTFL model in its current state is its inability to provide a clear mechanistic explanation for how the robust, approx. 24 hour periodicity of the system arises. Negative gene expression feedback loops are common mechanisms of mRNA homeostasis that are ubiquitous in cell biology. They are often employed in the propagation and regulation of signal transduction networks, e.g. NF-kB (oscillates with a period of around 2–3 h) and ERK signalling pathways (approximately 3–4 h) [47,48]. These cycles usually take just a few hours to complete, reflecting the time required for transcription, RNA processing, translation and repression/inactivation to occur and rapidly damp back to steady state within a few cycles. This is facilitated by rapid turnover of the repressive factor following its activation, usually via phosphorylation of phospho-degron sequences that direct the substrate for ubiquitin-mediated degradation at the proteasome [49].

In the mammalian circadian TTFL, the time constants associated with simple transcriptional feedback of this type are not sufficient to explain the day-long interval between the onset of transcription of the *Per* and *Cry* genes, mRNA translation, assembly, then the disassembly and degradation of their inhibitory complexes and components, respectively. Instead, a feedback loop model that considers only transcription and translation with no further regulation at the translational or post-translational levels could plausibly take no more than 3–4 hours to complete [50]. Therefore, post-transcriptional mechanisms particularly the post-translational regulation of PER and CRY activity are thought to play crucial roles in generating the delay in the negative arm of the feedback loop [51–53].

In the most broadly understood TTFL model, it is implied that rhythmic cellular functions are achieved via rhythmic clock-controlled gene transcription that drives rhythmic abundance and activity of the encoded protein. However, numerous studies have highlighted marked discrepancies between the cycling transcriptome and the cycling proteome. For instance, half of the oscillating proteins in mouse liver do not have a corresponding cycling mRNA [54] and in bone marrow-derived macrophages only 15% of detected rhythmic proteins have corresponding cycling mRNAs [29]; this trend extends to other eukaryotes [55–57]. Furthermore, unlike transcriptomic variation, the extent of daily proteomic variation is very similar when tissues from wild-type and TTFL-deficient mice are compared [58,59]. More significantly still, and irrespective of tissue/cellular context, the number and amplitude of cycling transcripts is generally far greater than that observed at the protein level [58,60]. Indeed, besides short-lived transcription factors, the abundance of most cellular proteins show little functional variation over 24 hours, perhaps because this would adversely perturb protein homeostasis that is essential for cell function and viability [61].

The question of how the TTFL model attains and maintains approx. 24 hour periodicity becomes yet more pressing when considering the mounting experimental evidence suggesting that rhythmic expression of core TTFL components may not be essential for circadian timekeeping at all. For instance, circadian rhythms have been observed in the complete absence of transcription, in red blood cells lacking nuclei [62], when the nucleus of the macroscopic alga *Acetabularia* has been removed [63,64], as well as in the alga *Ostreococcus tauri* where post-translational rhythms persist under constant darkness, even though transcription shuts down [65]. Moreover, mammalian fibroblasts and SCN tissues lacking a functional TTFL, via double knockout of TTFL genes *Cry1* and *Cry2,* retain circadian rhythmicity in a range of post-transcriptional processes [66]. It must be noted that circadian rhythms in cells/tissues where the TTFL has been genetically compromised are more variable (less robust) than their wild-type controls, however [66,67]. Evidently TTFL gene transcriptional feedback is important, but is not essential for rhythm generation or its consequences.

Taken together, these studies call into question the ability of the mammalian TTFL model, in its current form, to fully account for circadian timekeeping and physiology. How might the model be refined? Several groups have independently proposed the existence of a ‘cytoscillator’—a post-translational timing mechanism responsible for driving circadian coordination of cell function by modulating clock protein activity over the daily cycle [68–71]. This idea has an obvious precedent in the circadian organization of the prokaryotic cyanobacterium *Synechococcus elongatus* [72]. Here, a post-translational oscillator (PTO) is necessary and sufficient to account for the generation and period of the rhythm, but its robustness and physiological consequences arise through close coupling with a TTFL that lacks inherent daily periodicity [73]. The cyanobacterial PTO mechanism consists of three Kai proteins, Kai A/B/C, and is based around the rhythmic phosphorylation of KaiC and subsequent complex formation with the other two components, mediated by ATP hydrolysis. This system can be elegantly reconstituted *in vitro* with only the purified proteins and Mg-ATP, its circadian oscillations persist for weeks in constant conditions and are temperature compensated [74,75]. The existence of a mammalian equivalent has been speculated upon [53], and it is plausible that coupling of a circadian PTO harmonic oscillator (robust period, but sensitive to perturbation) with a TTFL relaxation oscillator (variable period but robust amplitude) in mammalian cells would allow many experimental inconsistencies and disparities to be resolved (figure 1). In this vein, it is interesting to consider post-translational modes of circadian regulation and its broad conservation in clock function. Components of a putative mammalian PTO could only definitively be established by reconstitution, as with cyanobacteria, but would likely include well-conserved and essential regulators of clock protein activity and circadian periodicity such as CK1, CK2, glycogen synthase kinase 3 (GSK3), the proteasome mediated degradation pathway, as well as protein phosphatases [53,68,76,77].

## 4. The discovery, diversity and importance of PERIOD genes

The first circadian mutants ever described were generated in *Drosophila melanogaster* by Ronald Konopka and Seymour Benzer, who identified three mutants in fly behaviour: an arrhythmic mutant, a short-period mutant with a period of approx. 19 hours and a long-period mutant of approx. 28 hours, all of which were mapped to the same locus [78]. They named this gene ‘*Period*’. Although misleadingly simple, this piece of work marked the beginning of the quest to unveil the molecular mechanisms behind circadian rhythmicity and provided the earliest example that single genes could control something as complex as animal behaviour, which was revolutionary at the time. The *Drosophila Period* gene was eventually identified and cloned 13 years later [79–81]; however, its function remained unclear. It took several more years until it was shown that *Period* mRNA cycled in *Drosophila* brains [42], which led to the hypothesis that a negative feedback loop involving PER protein lies at the core of the circadian clock mechanism.

The *Period* genes take different forms across different organisms. *Drosophila* has one *Per* gene, zebrafish have four *Per* genes, while mammals have three *paralogs*, namely *Per1*, *Per2* and *Per3*. In all these organisms, *Per* is essential for sustaining circadian rhythmicity. For instance, in mice, *Per1* and *Per2* are partially redundant with each other, but deleting both completely abolishes circadian rhythmicity [82,83]. In contrast, *Per3* knockouts show only modest circadian phenotypes that have led most to conclude its roles are modulatory, or context-dependent or facilitate other (non-circadian) cellular functions [51,82]. *In vivo* and in cultured cells, PER1 and PER2 proteins have short half-lives (<4 hours), with abundances that show the most robust circadian abundance rhythms across any TTFL components [84,85]. Cells that lack PER1/2, or highly overexpress PER2, do not show circadian regulation of *Per2* promoter activity [86,87]. Taken together, these observations have been used to support the idea that rhythmic expression of PER, either through rhythmic transcription or the resulting rhythmic PER protein abundance occurring due to daily variation in protein synthesis and stability [66], is the essential feature that drives circadian clock function in mammals. However, evidence has come to light suggesting there is greater nuance to the critical role of PER in the circadian mechanism and that regulation of its activity, rather than simply variations in its abundance should be investigated further. To begin exploring this and how it might refine our understanding of circadian timekeeping, we will first consider what is known about the physical features of the PER protein and how this regulates its interaction with other components of the cellular circadian machinery.

## 5. The organization of the PERIOD protein

**Figure 2.**
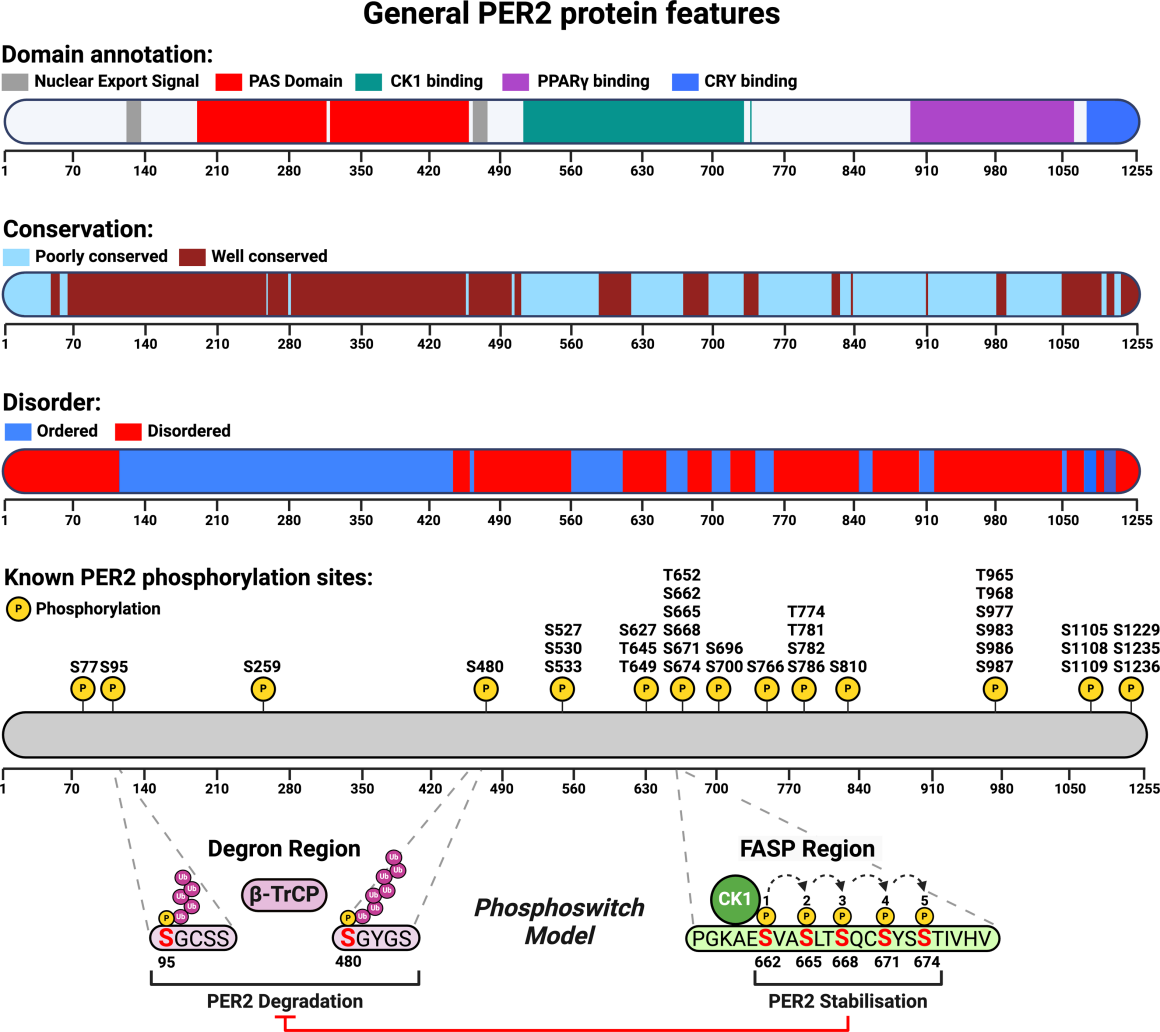
Schematic illustration of general PER2 protein features. Schematic of the human PER2 protein sequence annotated with its most well-characterized domains, relative residue conservation calculated via ConSurf, sequence disorder analysis calculated by CAID and known phosphorylation sites compiled from literature and PhosphoSitePlus. CK1δ/ε binds the CK1-binding domain on PER2 and can stabilize PER2 via phosphorylation at the FASP region, or facilitate degradation through phosphorylation in the degron region at S480 and, to a lesser degree, at S95, by promoting recruitment of the E3 ubiquitin ligase β-TrCP. The balance of phosphorylation between the FASP and the degron region determines overall PER2 stability and constitutes the phosphoswitch model. Figure created with BioRender.com.

Given their largely overlapping molecular characteristics and functions, PER1 and PER2 have often been considered perfect functional paralogs, although more recent work has begun to distinguish the differences in biochemical parameters and circadian phase of protein expression [85]. To date, PER2 has received the most attention due to stronger mutant phenotypes and the generation of the PER2::LUCIFERASE fusion reporter mouse two decades ago [23,88]. Given the deeper research background available, we will therefore focus on PER2 in this review, unless otherwise stated. PER2 is a very low abundance protein, translated from very low abundance mRNA, with OpenCell estimating the presence of 600–700 protein copies per cell [66,89] and 4–5 transcripts per cell [90]. The large overall size of PER2, approx. 135 kDa, lends itself to multiple protein interaction domains and sites of post-translational modification. A broad summary of some of these features is highlighted in figure 2.

## 6. The PAS domains

The N-terminal region of PER contains two PER-ARNT-SIM (PAS) domains (figure 2). PAS domains are found in a range of proteins of diverse function [91] and are often involved in sensing, signalling and most crucially, protein–protein interactions. The name of the PAS domain family comes from the proteins it was initially observed in, period (PER), aryl hydrocarbon receptor nuclear translocator (ARNT) and single-minded (SIM) [92]. Most eukaryotic proteins involved in the clock mechanism contain PAS domains: the basic-helix-loop-helix-PAS domain containing transcription factors CLOCK and BMAL activate the transcription of the PAS domain-containing transcriptional repressor PER. The tandem PAS domains, referred to as PAS-A and PAS-B, are made up of a series of α-helices and β-sheets, which adopt an ordered, globular fold that is highly conserved across species. In mice, the PAS-B domains of PER1/2/3 have been shown to mediate their homo/hetero-dimerization and their subsequent nuclear import [93–95]. Deletion of residues 348–434 in the PAS-B domain of PER results in shortened, approx. 22 hour period rhythms of locomotor activity in mice under constant darkness that eventually disintegrate to incoherence [96]. Moreover, the single point mutation W419E, located in the PAS-B domain of mouse PER2, significantly disrupts its ability to homo-dimerize [97]. Intriguingly, the analogous mutation in PER1 largely abolishes its phosphorylation by CK1δ [98]. Overall, the PAS domains of PER represent a highly conserved and critical component for its circadian function.

## 7. The casein kinase 1 binding domain

C-terminal to the PAS domains we find the CK1 binding domain. The interaction between PER and CK1 has been studied in molecular detail and adds a significant level of regulation to PER activity. In both mice and humans, CK1 is the major kinase responsible for phosphorylating PER paralogs [99–101]. Of the isoforms of CK1, δ and ε have been shown to be involved in clock function, with the δ isoform playing a larger role [102,103]. As a possible explanation for this, recent work has also shown CK1δ, but not *ε*, of being capable to phosphorylate CLOCK to promote CLOCK·BMAL1 removal from DNA during the early repressive phase, demonstrating a second role for CK1 in TTFL regulation [34,104].

The most extensively studied circadian substrate of CK1, however, is PER [99–101]. Phosphorylation by either CK1δ or ε is critical for modulating PER function by modulating its stability and localization [105–108]. Although PER has been shown to be phosphorylated by other kinases such as CK1α and γ, CK2 and GSK3β [109–112], the strongest evidence currently available suggests that CK1δ and ε are the major PER kinases. For instance, CK1δ/ε-deficient fibroblasts have a severely compromised molecular clock and reduced PER phosphorylation [113]. Pharmacological and genetic evidence suggest that changes in PER phosphorylation kinetics, stability and localization are set by the balance between CK1δ/ε and protein phosphatase 1 (PP1) and 2 A (PP2A) activity, which ultimately influences the period and phase of circadian oscillations [113–115].

Unlike typical kinase-substrate interactions which tend to be transient, CK1 forms a tight interaction with PER1 and PER2, binding stably throughout the cycle [104]; this is not the case for PER3 [51,105]. Following binding, CK1 is proposed to regulate the stability of PER through a phosphorylation-based mechanism, referred to as the ‘phosphoswitch’. Here, CK1 is able to phosphorylate two antagonistic sites on PER. The first of these, a degron located at S480 in humans (S478 in mice) leads to the proteasomal degradation of PER2, mediated by the E3 ligase β-transducin repeat-containing protein [106,116,117]. Conversely, phosphorylation at the second site, a serine cluster starting at S662 on PER2 in humans (S659 in mice), a region known as the Familial Advanced Sleep Phase (FASP) site, prevents phosphorylation of the degron, thereby stabilizing PER [99], with the balance of phosphorylation between these two sites predominantly determining the stability of PER (figure 2). Accordingly, the S662G polymorphism at the first serine residue in the FASP site leads to the destabilization of PER2, a shortening of circadian period, and a characteristic FASP site sleep/wake alteration in humans characterized by persistent early evening sleep onset and very early awakenings [118]. Recent work suggests the existence of an additional N-terminal phosphodegron located at S95 in humans (S93 in mice), that appears to act in concert with that around S480, albeit with more of a back-up role [119]. This site plays a more substantial role in PER1, which lacks the S480 degron site. The interaction between PER and CK1 is reciprocal, as phosphorylation of the serine residues in the FASP site results in their docking into a conserved anion binding site in close proximity to the active site of CK1, resulting in product inhibition [120].

The stability of PER, modulated by its phosphorylation, has been proposed to be the main regulator of circadian period [116]. However, possible evidence against this comes from the fungal clock model *Neurospora*, where it was clearly shown that the stability of FRQ (functionally analogous to PER) normally correlates with, but can be decoupled from, circadian period determination [121]. Additional evidence suggests phosphorylation via CK1 to be the main determinant of period, rather than FRQ stability [122]. Whether this is also true for mammalian PER remains to be definitively established, but it is notable that constitutively abundant PER does not abolish circadian rhythms *in vitro* or *in vivo* [44,51,123].

## 8. The CRY binding domain

At the extreme C-terminus of the PER, the CRY binding domain (CBD) mediates the interaction of PER with its best-known interacting partner, CRY. This is achieved through the PER CBD wrapping around the Photolyase Homology Region (PHR) of CRY [124]. In doing so, PER stabilizes CRY, as its CBD competes with F-box and leucine-rich repeat protein 3 which normally targets CRY for degradation [125,126]. CRY has two isoforms, CRY1 and CRY2, which have been suggested in the past to play different roles in the molecular mechanism of the TTFL, as shown in the case of *Cry1^-/-^* mice which have a short period, while *Cry2^-/-^* animals have a long period [127,128]. *In vitro*, CRY1 binds more tightly than CRY2 to CLOCK·BMAL1, this difference being attributed to a flexible serine loop adjacent to its PHR pocket [129]. However, the binding of PER2 CBD to CRY2 induces a conformational change in the latter, remodelling its ordered serine loop and thereby enhancing its affinity for CLOCK·BMAL1 [130]. In effect, the binding of PER2, via its CBD, to CRY2 strengthens the affinity of the latter for the transcriptional complex, similarly to CRY1 levels. This suggests that PERs have the ability to fine-tune the repressive power of CRYs and highlights the crucial role of the PER·CRY interaction in the TTFL circuit.

Another important aspect of the PER·CRY interaction to consider is their nuclear import. Early data suggested that mammalian PER and CRY proteins function similarly to their functional analogues in *Drosophila*, whereby they would accumulate as complexes in the cytosol and CRYs would license the nuclear translocation of PER [131], the timing of nuclear import being critical for correct circadian timekeeping [132]. However, while CRY binding does seem to stabilize PER against degradation [85], the nuclear translocation of PER has been shown to occur in the absence of CRY [94]. Instead, PER hyperphosphorylation has been suggested as the major factor that licenses PER nuclear entry [85]. The nuclear entry of PER is clearly required for formation of the early repressive complex. It is worth noting, however, that the copy number and half-life of PER is significantly lower than that of BMAL1, CLOCK or CRY1/2 (all >2000 copies per cell, Open Cell) [86]. To potentially explain this >two-fold difference in abundance, it has been suggested that PER in cells may not exist in the same early repressive complex until it is degraded, but instead may dissociate after removal of CLOCK·BMAL1 from DNA and move to a new CLOCK·BMAL1 target [133].

## 9. PERIOD is an intrinsically disordered protein

The majority of proteins must adopt a defined three-dimensional structure that defines their function. However, over one-third of eukaryotic proteins contain regions that almost entirely lack defined secondary structures [134], yet they are fully functional [135]. Protein segments lacking an apparent structure are referred to as intrinsically disordered regions (IDRs), and proteins mostly composed of such regions are referred to as intrinsically disordered proteins (IDPs). IDRs tend to be deficient in hydrophobic, bulky amino acids, thus preventing them from forming tightly packed hydrophobic cores that facilitate globular structure formation [136,137]. This leads to IDRs having different functions compared to structured regions. Mammalian TTFL proteins contain high levels of disorder, with the PER proteins standing out as the most heavily disordered, with around 60% of their sequence predicted to be disordered by the Critical Assessment of protein Intrinsic Disorder prediction (CAID) algorithms [138].

**Figure 3.**
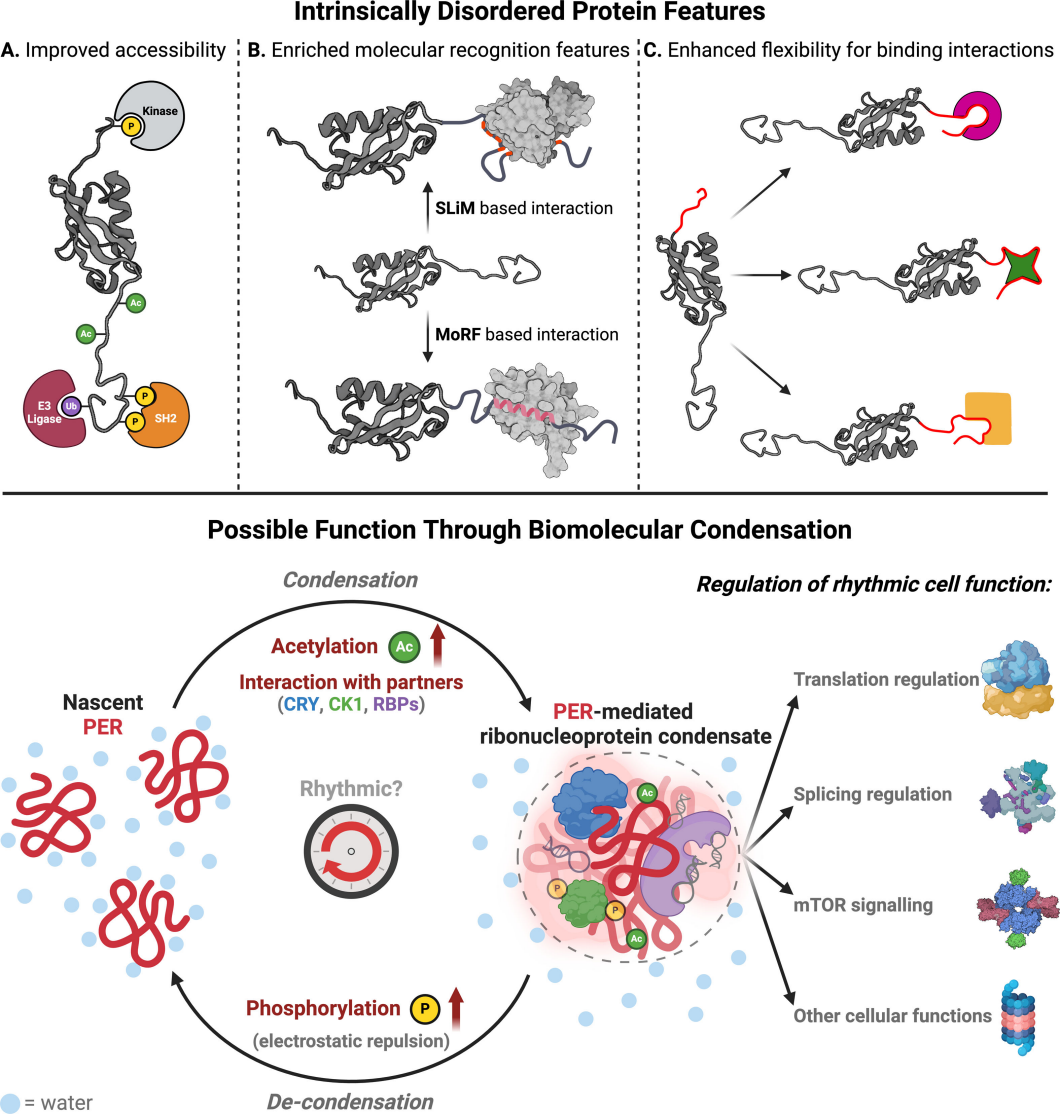
Schematic representation of properties associated with intrinsically disordered proteins and how they relate to PER function. The flexibility of intrinsically disordered regions improves their accessibility to proteins involved in the addition or removal of post-translational modification. Additionally, the availability of molecular recognition features such as SLiMs or MoRFs mediates the recruitment of a variety of binding partners through multivalent low-affinity interactions, thereby enhancing the network of protein–protein interactions available to IDR-containing proteins. The conformational flexibility and adaptability of disordered regions enable conformational plasticity at binding interfaces. Given the IDP features of PER, we hypothesize a model of PER activity through biomolecular condensation: the disordered conformation of nascent PER renders it unfavourable for hydration as an extended polypeptide, driving the adoption of more compacted states through biomolecular condensation that is modulated by homo-/heteromeric interaction with partners and favourable post-translational modifications, i.e. those which reduce the overall charge on PER, such as acetylation. Dissociation of PER and partners from condensates may be driven through electrostatic repulsion generated through progressive phosphorylation, by CK1 for example. PER-containing ribonucleoprotein condensates would therefore be perfectly positioned to impart circadian regulation to cell function. This testable model may provide a basis for understanding rhythmic PER activity, that we predict would be amplified and reinforced by feedback via canonical TTFL-mediated changes in PER production. Figure created with BioRender.com.

Perhaps counter-intuitively, lacking a defined structure can confer several advantages on a protein [139,140]. The absence of a confined structure facilitates a larger surface area for interaction and offers a much higher degree of conformational flexibility. This is achieved through a variety of peptide motifs that enrich the potential interactome of IDPs and make the regulation of their function, stability and localization more fine-tuneable via post-translational modifications [141]. As such, the dynamic spectrum of conformation states associated with IDRs makes them favourable components of proteins which need to coordinate complex regulatory events in space and time through recognition of specific interacting partners [142], such as the role of PER in the circadian clock. IDR function can be classified into three main categories according to their interaction features (figure 3). First, the openness and flexibility of IDRs improves their accessibility to enzymes that add or remove post-translational modifications (PTMs) and that of proteins which bind these PTMs [143]. For instance, changes in phosphorylation and ubiquitination of IDPs are important for modulating their activity and stability via the resulting changes in cellular localization or protection from the cellular degradation machinery [142]. We have previously discussed the intricate phosphorylation-mediated regulation of PER stability that occurs via the phosphoswitch mechanism. However, PER is phosphorylated outside the phosphoswitch, as well as undergoing PTMs besides phosphorylation [52]. For instance, O-GlcNacylation occurs at the first serine in the FASP site in competition with phosphorylation, in a partly glucose-mediated manner, thereby working as a metabolic sensor for TTFL regulation [144]. Intriguingly, the transferase responsible for O-GlcNacylation also comes under the regulation of GSK3β, which we have previously suggested to be part of the PTO model (figure 1) [53,110,145]. Additionally, PER2 acetylation, through mechanisms not yet fully elucidated, has been implicated in regulating PER stability [146] by competing with ubiquitination for substrate lysine residues and by modulating CK1 activity at the FASP site [147].

Second, the availability of vast arrays of linear motifs and molecular recognition features (MoRFs) within IDRs enables the scaffolding and recruitment of different binding partners. IDRs are often rich in short linear motifs (SLiMs), 3–10 amino acid long stretches which mediate low-affinity interactions. They primarily bind to globular domains, often multiple times on the same protein, and can promote complex formation, target proteins to specific subcellular localizations or recruit enzymes that can change the nature of the motif itself via PTMs [143]. Examples of SLiMs include kinase recognition motifs such as the cyclin-dependent kinase recognition motif [ST]Px[KR] [148], complex promoting motifs such as PxxP motifs that interact with SH3 domains [149] or nuclear localization signals made up of short clusters of K/Rs [150]. The Eukaryotic Linear Motif is a useful online resource for predicting SLiMs [151] and has been used to investigate SLiMs in mammalian PER and FRQ. These data highlighted the ubiquitous localization of SLiMs for validated interactors in regions of disorder [152]; however, a clear demonstration of the functional importance of these newly identified SLiMs has yet to be established. Additionally, IDRs can contain MoRFs, spans of 10–70 amino acids that undergo disorder-to-order transitions when binding and promoting specific interprotein interactions [153–155]. The oncoprotein p53 is a prime example, as its first N-terminal MoRF transitions from a disordered state into an α-helix upon binding to MDM2 and is known to interact via MoRFs with close to 40 different proteins [154]. This latter point additionally highlights the third category of IDR function, as the conformational flexibility and adaptability of disordered regions enable them to mould into almost perfect fits for the binding interfaces of various interacting proteins, with vastly different outcomes, as drastic as inhibiting signalling processes in one instance while activating them in another [140,156].

Although disordered protein regions often gain structure upon binding to an interacting partner, IDP-containing complexes frequently retain considerable conformational freedom, preserving disordered state features. Regions that go through disorder-to-order transitions can undergo further unfolding/refolding, covering a spectrum of states that range from dynamic to static. These IDP complexes are often referred to as ‘fuzzy’ complexes [157,158]. As IDPs, TTFL proteins across species are predicted to make use of the multitude of features described above. Indeed, these types of features have been previously described to be involved in the function of PER/FRQ. Here, conformational changes in the structure of their IDRs are thought to govern specific time-of-day-dependent interactions with a variety of partners, which are involved in the post-transcriptional regulation of the clock mechanism [152]. For example, the interaction of FRQ with its major interacting partner, FRQ-interacting RNA helicase, was shown to be mediated by a specific set of SLiMs, as well as positively charged patches that mediate electrostatic interactions forming a ‘fuzzy’ complex involved in the negative arm of the TTFL feedback loop [159]. Similarly, the largely disordered C-terminus of PER2 undergoes disorder-to-order transition upon binding to CRY in mouse, via MoRFs constituting its CRY-binding domain [124,160].

## 10. Function through biomolecular condensation

The cellular environment is extraordinarily dense [161]. Through their high concentration in the cytosol, macromolecules restrict the movement and access of other molecules to certain volumes, binding interactors and crucially, to water [162]. Macromolecular crowding at concentrations found in the cytoplasm (350–550 mg ml^−1^) profoundly reduces the thermodynamic favourability of protein hydration compared with dilute solutions and favours macromolecular assembly through multimerization and aggregation, that minimize the proportion of ‘structured’ water in solvation shells [163,164]. While solvent thermodynamics ultimately drive all protein folding and macromolecular interactions to maximize the availability of ‘free’ water and optimize its network of hydrogen bonds, the entropic cost of hydration is readily offset by enthalpically favourable electrostatic interactions, e.g. at charged residues [164,165]. This is equally true for metastable IDRs and IDPs as it is for structured domains with defined global energy minima.

Macromolecular crowding and the energetic cost of macromolecular hydration can stimulate liquid–liquid phase separation (LLPS), a density transition whereby dispersed proteins assemble into dense droplets. In cells, these are variously referred to as membrane-less organelles (MLOs), ribonucleoprotein bodies/granules or biomolecular condensates [163,166]. The link between molecular crowding, phase separation and cellular signalling is beautifully showcased by the activity of with-no-lysine kinases, which undergo phase separation in response to hyperosmotic stress-induced crowding to signal the activation of downstream effectors [167]. Abundant evidence highlights biomolecular condensation as an essential strategy for spatiotemporally organizing the intracellular environment and coordinating its vast array of reactions [168]. While a direct link between central TTFL components, phase separation and circadian regulation is yet to be comprehensibly described, an emerging example is showcased by the RNA-binding proteins ATAXIN2 and ATAXIN2-Like which are suggested to form spatiotemporally coordinated condensates that can rhythmically recruit specific mRNA transcripts for translation of TTFL components, including PER2, in mammals [169]. Similarly, work in *Neurospora* describes the clustering of *Frq* mRNAs near nuclei via interaction with an RNA-binding protein [170]. Recent work also suggests that while overexpressed PER does display the capacity for LLPS mediated by weaker electrostatics, endogenous levels of PER predominantly form more stable ‘microbodies’, likely mediated by both electrostatic and stronger protein–protein interactions [171]. An additional example includes a description of FRQ possibly engaging in phase separation *in vitro*, thereby recruiting CK1 and modulating its activity [172].

In cells, IDPs such as PER are typically driven to adopt more compact states by forming protein complexes and condensates with various interacting partners [173]. A prime example of this occurs during ribosome assembly, which relies on the sequential assembly of RNA and numerous disordered ribosomal protein subunits [174,175] that adopt their final stable fold only upon complex formation. The IDP features of PER implicate it as scaffold protein, capable of coordinating complex regulatory events in space and time. Large PER-mediated complexes have been certainly described in mouse [104,176]; however, a structure or a detailed understanding of the components present in such a complex and their stoichiometries remains elusive at this time.

Although some IDRs alone are sufficient to promote phase separation in solution, in cells, protein-RNA interactions also appear to be central to this phenomenon. PER does not contain any canonical RNA-recognition motifs; however, its many potential SLiMs and MoRFs could facilitate multivalent interactions with other MLO components. The composition and integrity of MLOs are not fixed, but are highly susceptible to variations in the cellular environment, including changes in osmolarity, temperature, ionic strength and local protein concentration [164,177]. Changes in surface electrostatics via PTMs, particularly phosphorylation, are also crucial to consider [164,178]. For example, PER hyperphosphorylation would be expected to increase the relative favourability of hydration and/or electrostatic repulsion from other MLO components, which would facilitate egress from MLOs while favouring other interactions [143]. If correct, then the interactome of PER is likely to vary across the circadian cycle, as its propensity to undergo phase separation is expected to change with macromolecular crowding and PTMs, as well as its abundance. Circadian variation in cytoplasmic macromolecular crowding [28] and PER hyperphosphorylation [87] have both been described and might plausibly contribute to temporal variation in its cytosolic sequestration and subsequent assembly into higher order complexes.

## 11. Conclusions: rhythmic transcription, abundance or activity?

The most broadly understood TTFL model, in its current state, is reliant on two main pillars to account for circadian timekeeping: the rhythmic transcription/transcript abundance of *Per* genes and the resulting rhythmic PER protein abundance. Studies performed in a wide range of model organisms have asked the question of whether rhythmic transcription of *Per* (or its functional equivalents) is essential for rhythmicity. In *Drosophila*, constitutive expression of *Per* RNA did not abolish circadian rhythms in behaviour [179], and constitutive expression of *Per* via arrhythmic promoters restored rhythmicity in *Per* null flies [180,181]. Similarly, rhythmic behaviour in *Per1^−/−^/Per2^−/−^* double knockout mice can be restored via constitutive *Per2* expression, further emphasizing the non-essential role of rhythmic *Per* transcription for circadian rhythms in mammals [86].

Various studies have also sought to address whether rhythmic PER protein abundance is indeed essential for rhythmicity. Constitutive overexpression of FRQ protein eliminated circadian rhythmicity in conidiation in *Neurospora* [40]. Comparably, Chen *et al.* showed that constitutive overexpression of PER1 and PER2 abolished rhythmicity in PER protein rhythms as well as in that of other TTFL genes mRNAs, yet preserved rhythms in PER·CRY and CLOCK·BMAL1 interaction [87]. However, work undertaken by Okamura *et al.* in mouse cells showed directly contradictory evidence, as constitutive PER2 overexpression did not abolish rhythms in *Per2* mRNA or the mRNA of *Dbp* [44]. More recently, the circadian clock was reported to function independently of PER abundance rhythms in mouse liver [51], further suggesting that rhythmic PER protein abundance is not essential for circadian physiology or TTFL activity in mammals. As an alternative, the stability of PER, as modulated through previously discussed PTMs (phosphorylation, O-GlcNacylation, acetylation and ubiquitination), has been proposed to be the main regulator of circadian period, given many observations indicating that an increased rate of degradation leads to period shortening and vice versa [102,182]. However, despite this apparent correlation, the stability of both PER and CRY can apparently be decoupled from circadian clock function [51,182,183].

Taken together, constitutive *Per* transcription as well as constitutively expressed PER protein are compatible with sustaining circadian rhythmicity and while PER phosphorylation correlates with PER stability, the latter does not appear sufficient to drive rhythmicity. Therefore, another facet of PER activity that is regulated post-translationally, for instance via phosphorylation, may ultimately be responsible for driving circadian TTFL rhythms and physiology more broadly. We suggest that elucidating the causal mechanisms that confer rhythmic activities (besides stability) onto PER proteins is the major challenge for circadian cell biology and protein biochemistry in the coming years. The development of post-translational reporters of circadian cell function that lie outside the TTFL genetic circuit would greatly aid this endeavour. Moreover, while purely genetic approaches to understanding circadian timing have been profoundly important for identifying relevant cellular components over the last half century, it is possible their utility may be approaching exhaustion because there is only so much that can be inferred about the behaviour of a dynamic system from perturbing its steady state.

PER is continuously synthesized and turned over and so it is plausible that upon production, its PTMs, activity, stability and localization are rapidly acquired through interactions with pre-assembled PER/CK1 complexes that reflect the state of the cell at that circadian phase (figure 3). Contrary to following a slow and stately procession each day, as currently believed, it is tempting to speculate that each PER molecule enters transiently into a daily dance of post-translational modifications and higher order assemblies. These could be conducted by a dynamic network of regulatory acetylases/deacetylases, kinases/phosphatases and modulated by changes in macromolecular crowding that drive PER activity, phase separation, its interactions, steady state abundance and localization to follow a circadian rhythm, which is reinforced by feedback repression, not generated by it. Perhaps it is the wave that moves, not the water.

## Data Availability

This article has no additional data.
